# Research progress of post-acute sequelae after SARS-CoV-2 infection

**DOI:** 10.1038/s41419-024-06642-5

**Published:** 2024-04-11

**Authors:** Taiwei Jiao, Yuling Huang, Haiyan Sun, Lina Yang

**Affiliations:** 1 Department of Gastroenterology and Endoscopy, The First Hospital of China Medical University, Shenyang, Liaoning 110001 P.R. China; 2 Department of Geriatrics, The First Hospital of China Medical University, Shenyang, Liaoning 110001 P.R. China; 3 Department of Endodontics, School of Stomatology, China Medical University, Shenyang, Liaoning 110001 P.R. China; 4 Department of International Physical Examination Center, The First Hospital of China Medical University, Shenyang, Liaoning 110001 P.R. China

**Keywords:** Molecular biology, Diseases

## Abstract

SARS-CoV-2 has spread rapidly worldwide and infected hundreds of millions of people worldwide. With the increasing number of COVID-19 patients discharged from hospitals, the emergence of its associated complications, sequelae, has become a new global health crisis secondary to acute infection. For the time being, such complications and sequelae are collectively called “Post-acute sequelae after SARS-CoV-2 infection (PASC)”, also referred to as “long COVID” syndrome. Similar to the acute infection period of COVID-19, there is also heterogeneity in PASC. This article reviews the various long-term complications and sequelae observed in multiple organ systems caused by COVID-19, pathophysiological mechanisms, diagnosis, and treatment of PASC, aiming to raise awareness of PASC and optimize management strategies.

## Facts

- PASC is very likely to affect the quality of life of the COVID-19 patient after “recovery”.
- After SARS-CoV-2 infection, it mainly affects respiratory, cardiovascular, neurological, psychiatric, urinary, blood, skin and digestive systems.
- It is generally believed that the persistence of low-load virus, abnormal immune response caused by viral proteins, secondary autoimmune diseases, and multisystem damage caused by microthrombosis and other factors lead to the occurrence and progression of PASC.

## Open questions

- What do we know about PASC?
- How should we manage PASC?
- Whether the recovered group will have new long-term sequelae in the future?

## Introduction

Corona Virus Disease 2019 (COVID-19) caused by the novel coronavirus severe acute respiratory syndrome coronavirus-2 (SARS-CoV-2) has caused extremely serious harm to human life and health since its outbreak at the end of 2019. Today, the number of “recovered” patients is increasing, and there is more and more evidence that a considerable proportion of patients who have been hospitalized for “recovered” due to COVID-19 have long-term complications and sequelae of multiple organs and systems [1, 2], including various physical and neuropsychiatric symptoms, which generally last more than 12 weeks [3, 4]. Although people’s understanding of the clinical manifestations and treatment methods of COVID-19 has begun to increase, little is known about the complications of COVID-19, the duration of sequelae, clinical manifestations, and risk factors. Previous studies have shown that patients with severe acute respiratory syndrome (SARS) and Middle East respiratory syndrome (MERS) have widespread systematically related persistent symptoms [5], while SARS-CoV-2 is associated with severe acute respiratory syndrome coronavirus (SARS-CoV) and Middle East respiratory syndrome coronavirus (MERS-CoV) belongs to the same family of coronaviruses, the genus Coronavirus β, has a similar viral structure [6], especially with SARS-CoV has a specific binding receptor Angiotensin-converting enzyme 2 (ACE2) [7], thus play a pathogenic role. In low- and middle-income countries, much attention is given to people who die or “recover” from the virus, while less care is given to those who “recover” and still have long-term symptoms. As a result, the term “rehabilitation” may be misused [8]. This study reviews the long-term clinical manifestations of systemic injuries caused by the SARS-CoV-2 infection to improve people’s understanding of post-acute sequelae after SARS-CoV-2 infection (PASC), also referred to as “long COVID” syndrome.

## Pathogenesis of SARS-CoV2

**Fig. 1:**
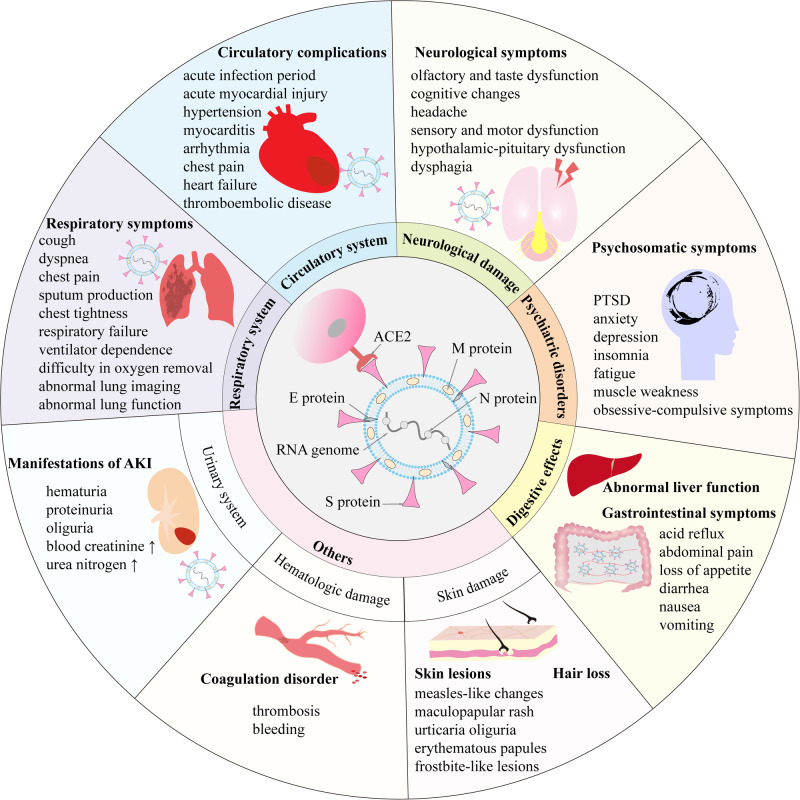
Pathogenesis of SARS CoV2 and clinical features of PASC.

As a new member of the genus Coronavirus β of the Coronavirus family [9], the single-stranded positive-stranded RNA genome of SARS-CoV-2 encodes four important structural proteins: spike surface protein (S protein), nucleocapsid protein (N protein), matrix protein (M protein), and small envelope protein (E protein). Among them, the spike protein S embedded on the surface of the viral envelope E protein is the key structure that determines the virus to invade the host cell, including two subunits, S1 and S2. The S1 subunit contains the receptor-binding domain (RBD), which is responsible for binding to the host recipient; The S2 subunit promotes membrane fusion between the virus and host cells [7]. The spike protein S enters cells by binding to specific ACE2 receptors on the surface of target cells [10], and eventually attacks a variety of target cells expressing ACE2, including vascular endothelium, heart, gastrointestinal tract, kidney, etc. in addition to the most important alveolar cells [11]. At the same time, damaged target cells such as alveoli release a large number of viruses and pro-inflammatory factors, further activate the immune system, release cytokines, and cause cytokine storms [12, 13], thereby further aggravating the condition, causing acute respiratory distress syndrome [14] and even multi-organ dysfunction syndrome [15] and so on (Fig. 1).

## Definition of PASC and its reasons

The World Health Organization points out that “long COVID” refers to symptoms 3 months after infection with the SARS-CoV-2, lasting at least 2 months, and the condition cannot be explained by other diagnoses, now been changed to “Post-acute sequelae after SARS-CoV-2 infection (PASC)”. These symptoms may be new after recovery from acute infection, or they may appear from the beginning of the disease and may recur, often affecting daily functioning. One cohort study [16] found that 26% of people still had moderate to severe symptoms 2 months after infection, and 15% developed moderate to severe symptoms at 8 months, with the most common symptoms being a loss of taste or smell, fatigue, and shortness of breath. Another study from Wuhan [17] conducted a follow-up of 1733 patients recovering from SARS-CoV-2 infection for 6~9 months and found that 76% of patients had at least one symptom after 6 months of onset, and the proportion of women was higher, the most common symptom was fatigue or muscle weakness, 26% had difficulty sleeping, 23% had anxiety or depression, and even after 6 months.

In addition to the direct attack of SARS-CoV-2 on the body, immune system disorders and inflammatory response disturbances caused by acute phase infection may be the pathophysiology of long-term sequelae for the causes of persistent injury in COVID-19-related devices [18]. Studies on major inflammatory markers such as neutrophils and C-reactive protein have shown that a low-grade inflammatory response persists after discharge from the hospital, causing oxidative stress and tissue damage [19]. In addition, an assessment of the long-term mental health of patients recovering from COVID-19 revealed significant post-traumatic stress disorder, anxiety, depression, insomnia, and other psychological health problems, which may be affected by a variety of factors such as gender, age, social stability, economic status, social isolation, and fear of infection [20].

## Clinical features of the PASC

Symptoms of PASC may include fatigue, muscle pain, weakness, and low-grade fever; cough, shortness of breath, and chest pain; headache, cognitive retardation; rashes, such as frostbite-like lesions, blisters, and maculopapular rashes; mental health problems, including mood swings; thrombotic diseases, etc. Some symptoms, such as fatigue, etc. may be constant, while others are intermittent (Fig. 1).

### Respiratory system damage of PASC

Although the SARS-CoV-2 infection can have a wide impact on the whole body, the lung as the focus of the virus attack, an acute infection caused by a variety of pathological changes can lead to lung parenchyma and interstitial damage through a variety of mechanisms, promote pulmonary fibrosis and lead to the occurrence of long-term symptoms [21], mainly including cough, dyspnea, chest pain, sputum production, chest tightness, etc. Some patients will have ventilator dependence and difficulty in oxygen removal, accompanied by lung imaging lung function abnormalities, and other manifestations of pulmonary fibrosis. Physiological changes caused by an acute infection of the virus can lead to pulmonary fibrosis and many long-term complications. Taking pulmonary fibrosis as an example, when the virus directly enters cells, especially type II alveolar epithelial cells that stabilize the epithelial barrier, it leads to cell death, which in turn leads to an increase in inflammatory cytokines. The resulting diffuse alveolar damage and inflammatory cytokines aggregate lymphocytes, macrophages, and neutrophils, which in turn aggregate fibroblasts and ultimately lead to pulmonary fibrosis [22].

A meta-analysis [23] showed that dyspnea, chest pain, and cough were the most common respiratory symptoms after SARS-CoV-2 infection, with an incidence of 37%, 16%, and 14%, respectively. Other studies [2] also showed that dyspnea was the most common, with 22.9~53.0% of patients still having dyspnea 2 months after infection. 6.6% of discharged “rehabilitation” patients require long-term oxygen intake [24]. At 3 months follow-up after discharge, 60% of patients developed a cough, 43% had increased sputum production, and 62% had post-exertive chest tightness and palpitations [25]. Patients who undergo tracheostomy due to respiratory failure still need long-term breather-assisted breathing after “recovery”, and it is difficult to get out of the machine. A multicenter study in Spain [26] showed that 1890 patients who underwent tracheostomy in 120 hospitals had only 52 patients after 1 month of follow-up. 1% of patients were successfully weaned.

Three months after infection, some patients develop radiographic abnormalities, including ground-glass changes and subpleural band-like high-density opacities, with abnormal lung function; six months after infection, some patients develop persistent CT changes, including ground-glass changes and ongoing progression suggestive of fibrosis, such as reticular formation and varying degrees of lung parenchymal damage [27]. In a retrospective study [28] of 52 patients with a three-month interval between the first CT and follow-up CT, 42.3% of whom had residual lesions on CT, most commonly ground-glass changes (54.5%), followed by mixed ground-glass changes and subpleural consolidation (31.8%) and pulmonary consolidation (13.7%), and these patients had higher severity scores on first CT, longer hospital stays, higher ICU admissions, and higher WBC counts. Studies [29] have also shown substantial improvement in CT in most patients 4 weeks after discharge, but persistent abnormalities are present in 54% of patients, most commonly with thickening of the lobular septa and focal or multisite ground-glass changes. In contrast, CT in critically ill patients requiring mechanical ventilation three months after discharge reveals normal imaging findings in only 4% of patients, ground-glass changes in 89%, and fibrosis in 67% [30]. When patients with severe COVID were “recovered” and discharged three months after discharge, 81% of patients still had abnormal chest CT imaging [31].

Approximately 75% of discharged patients develop abnormal lung function within 30 days, and 25% remain abnormal for 3 months after discharge, most commonly with decreased diffusion function of carbon monoxide, and decreased diffusion function and abnormal findings on CT and degree of fibrosis are associated with disease severity [32]. Another study showed that 3 months after discharge, about 55.7% of patients had chest CT abnormalities, mainly manifested as ground glass shadow (44.1%), while 44.3% of patients had abnormal lung function, mainly manifested as impaired lung diffusion ability (34.8%) [33], and these lung injuries continued to account for a large proportion of the six-month follow-up [17]. For critically ill and severely ill patients, persistent impaired lung function is more likely after discharge [34, 35], and a 6~9-month follow-up study of 1733 patients who recovered from SARS-CoV-2 infection [17] found that patients with more severe disease had an increased risk of pulmonary diffusion dysfunction, and the proportion of severe patients was higher. Therefore, long-term and close follow-up of patients diagnosed as critically ill and severe during acute infection is needed to facilitate early rehabilitation. A Chinese study of 55 non-critically ill patients with SARS-CoV-2 pneumonia found that during a three-month follow-up period, pulmonary function tests showed abnormalities in 25% of patients and decreased pulmonary diffusion in 16% [36]. A study by Salem et al. [37] yielded similar results.

A research team that conducted 3, 6, 9, and 12 months of continuous follow-up after discharge in 83 critically ill patients who did not require mechanical ventilation during hospitalization to assess their lung function, exercise capacity, and chest high-resolution CT revealed that the degree of dyspnea and exercise capacity of most recovered patients improved over time, although at 12 months of follow-up, 33% of patients still had impaired diffusion of pulmonary carbon monoxide (<80% predicted), chest HRCT is abnormal in 24% of patients, and women are a high-risk factor for persistent pulmonary diffuse dysfunction [38]. A follow-up study in Germany divided 180 patients into groups without hospitalization and hospitalized without oxygen, requiring low-flow oxygen, requiring high-flow oxygen, invasive mechanical ventilation, and receiving extracorporeal membrane oxygenation according to the severity of acute infection, and showed that the severity of acute infection was significantly associated with lung function impairment, chest CT, and respiratory symptoms at 1-year follow-up [39]. These findings suggest that a significant proportion of patients with COVID-19 have long-term imaging and lung function abnormalities after discharge. Of particular note is that a recent follow-up study of 71 healthcare workers who had been infected with SARS for up to 15 years showed that some patients still had varying degrees of lung CT and abnormal lung function [40]. It is speculated that lung damage in COVID-19 recovery patients may also last for a long time, and it is necessary to carry out relevant pulmonary function rehabilitation training to improve their lung function while continuing close follow-up. Consensus and guidelines have been issued in many countries proposing early post-discharge pulmonary rehabilitation measures for patients with COVID-19 [41]. For example, the pulmonary rehabilitation (PR) program [42] is a core component of the management of patients with chronic respiratory diseases. Multidisciplinary and personalized respiratory physiotherapy, endurance training, daily life training, psychological support, and other comprehensive measures can significantly improve the lung function, exercise ability, and quality of life of COVID-19 patients [43]. It has been proven to be effective, feasible, and safe for the respiratory sequelae of COVID-19 patients with different severity and severity grading [41] (Table 1).

**Table 1:** Respiratory system damage during follow-up of COVID-19 patient.

No. of patients	Age and sex	Patient characteristics	Follow-up time	Respiratory symptoms	Radiographic abnormality	Pulmonary function	Ref.
76	41.3 ± 13.8 years; 55 female (72%)	65 (86%) healthcare workers; no invasive mechanical ventilation	3-month	15 (20%) with a fever, 45 (60%) with a cough, 33 (43%) with increased sputum production, 47 (62%) with activity chest tightness and palpitations, 45 (60%) complained of fatigue	HRCTs returned to normal in 50/61 (82%) patients	42% of survivors had mild pulmonary function abnormalities	[25]
52	50.17 ± 13.1 years; 32 males (61.54%)		3-month	fever (46/52, 88.4%), fatigue (28/52, 53.8%), and dyspnea (21/52, 40.4%)	The most common radiological pattern on initial CT was GGO (24/52, 46.2%), followed by consolidation (14/52, 26.9%) and mixed pattern (14/52, 26.9%).	25.4% of the patients, mostly demonstrated diffusion reductions in DLCO	[28]
51	46.6 ± 13.9 years; male 21 (41.18%)	3 (5.9%) smokers	4-week	33.3% complained of clinical symptoms	CT scan showed lung abnormalities gradually resolved		[29]
48	63 (55–68) years; male 33 (68.8%)	All are mechanically ventilated survivors; 23 (48%) smokers	3-month		HRCTs showed ground-glass opacities in 89% of cases	Diminished TLC and diffusion capacity in 23 and 36 participants	[30]
91	66 (59–73) years; female 31 (34%)	All are severe COVID-19 patients; no patients were intubated	3-month	59 (65%) with dyspnea, 36 (40%) with asthenia, and 16 (18%) with cough	74 (81%) patients had CT abnormalities	Impaired DLCO in 33 (36%) and VA in 24 (26%) participants	[31]
57	46.72 ± 13.78 years; male 26 (45.6%)	40 non-severe cases and 17 severe cases; 9 (15.7%) smokers	1 month		31 (54.3%) patients had abnormal CT findings	Abnormal pulmonary function in 43 (75.4%) patients	[32]
1733	57 (47–65) years; male 897 (52%)	68% of participants required oxygen therapy during the hospital, and 7% required HFNC, non-IMV, or IMV; 4% were admitted to ICU; 102 (6%) smokers	6-month	1038 (63%) with fatigue or muscle weakness, 437 (26%) with sleep difficulties	Ground glass opacity and irregular lines were common HRCT pattern	pulmonary diffusion abnormality in 22–56% of participants	[17]
647	58 ± 15 years; male 285 (44%)	399 non-severe patients and 248 severe patients	3-month	87 (13%) with weakness, 63 (10%) with palpitations, 56 (9%) with dyspnea	CT scan showed patients with abnormal DLCO were more likely to have interstitial damage	abnormal diffusing capacity of DLCO in 44 (54%) patients	[34]
60	59 (27–82) years; male 43 (71.67%)	All patients requiring high flow nasal oxygen, non-invasive or invasive ventilation; 3 smokers	3–6 months			Impaired lung function with reduced DLCO in 52% of subjects	[35]
55	47.74 ± 15.49 years; female 23 (41.82%)	14 patients requiring additional oxygen therapy; no one requiring mechanical ventilation; 4 smokers	3-month	14.55% with exertional dyspnea, 1.81% with cough and sputum	Radiological abnormalities were detected in 39 patients	Lung function abnormalities in 14 patients	[36]
83	60 (52–66) years; male 47 (57%)	37 (45%) nasal cannula or mask; 46 (55%) HFNC or NIV	3-month, 6-month, 9-month, and 12-month	The number of patients with dyspnea reduced at 6 months, 9 months, and 12 months, 4 (5%) patients with persistent dyspnea at 12 months	Radiological changes persisted in 20 (24%) patients	A significant reduction in DLCO	[38]
180	56.50 (43.25–65.75) years female 68 (37.78%)	62 (34.44%) smokers	12-month	60.87% with fatigue, 43.48% with shortness of breath	The CT score increased with acute severe COVID-19	median TLC, FVC, DLCO, and KCO increased up to month 6, with no further improvement between months 6 and 12	[39]

### The effect of PASC on the circulatory system

SARS-CoV-2 can bind to angiotensin-converting enzyme 2 (ACE2) receptors located on the surface of cardiomyocytes, which is also the route for the virus to enter cardiomyocytes [10]. In a single-cell RNA sequencing study, ACE2 receptor expression was present in 7.5% of cardiomyocytes, putting them at increased risk of being a target organ for direct viral injury [44]. This was confirmed by autopsy studies, in which 61.5% of the dead patients had coronavirus RNA in their myocardium [45]. The mechanism of cardiovascular system disease damage after SARS-CoV-2 infection is not fully understood, and possible mechanisms include persistent damage caused by direct invasion of cardiomyocytes by viruses and apoptosis in the later stage, inflammation caused by endothelial cell infection, transcriptional changes in various cell types in cardiac tissue, complement activation and complement-mediated coagulation dysfunction and microangiopathy, downregulation of ACE2 and dysregulation of renin vasotensin aldosterone system, autonomic dysfunction, elevated and passing of pro-inflammatory cytokines The activation of TGF-β signaling by the Smad pathway induces cardiomyocyte fibrosis and scar tissue formation, and the abnormal persistent immune activation state, autoimmunity or the persistence of the virus at the site of immune activation are also considered to be possible causes of extrapulmonary manifestations after SARS-CoV-2 infection [46, 47].

The direct and indirect mechanisms such as SARS-CoV-2 attack and cytokine storm, ischemia, and hypoxia lead to damage to cardiomyocytes and vascular endothelial cells, resulting in a series of circulatory complications in the acute infection period, mainly including hypertension, acute myocardial injury, myocarditis, arrhythmia, etc. [17], among which myocardial injury is obviously related to the fatal outcome of the acute infection phase in the hospital [48]. Studies have shown that cardiovascular symptoms can persist until after hospital discharge, while the new-onset cardiovascular disease may develop. A cohort study in Wuhan showed that 13% of COVID-19-recovered patients developed significant cardiovascular symptoms, including increased heart rate and newly diagnosed hypertension, 3 months after discharge [49]. Carfì et al. [2] described that up to 21% of patients experienced chest pain discomfort within 60 days of discharge. At a follow-up of 60 days, palpitations occurred frequently in 9% of patients. It has been reported [50] that the incidence of orthostatic tachycardia increases after human infection with SARS-CoV-2. In addition, a routine examination of 100 patients with COVID-19 who had been discharged from the hospital found that troponin T was elevated in 60% of patients, and nearly 71% of them developed long-term myocarditis, this finding has been controversial in clinical care because other similar studies have not been reported, but it also seems to indicate that the effect on the heart is prolonged after infection with SARS-CoV-2 [51]. In a study of 1216 hospitalized patients with COVID-19 [52], 55% of patients had abnormal echocardiograms, including 46% of 901 patients without prior heart disease. Although echocardiographic results are not published at subsequent long-term follow-up, cardiac complications during hospitalization must also be taken into account in the long-term care of these patients.

A follow-up of 139 medical staff who had COVID-19 showed that 11 weeks after discharge, 41.7% of recovered patients had at least one symptom related to the cardiovascular system, mainly manifested as chest pain, dyspnea, etc., 49.6% of participants had ECG abnormalities, and 60.4% of recovered patients had cardiac function magnetic resonance imaging (CMR) abnormalities, which were shared 30.9% of participants with CMR now meet the criteria for pericarditis and/or myocarditis [53]. CMR is a non-invasive reference standard for cardiac function and tissue characterization of impairment, and in patients with confirmed or suspected active COVID-19 and clinical evidence of myocardial injury, CMR provides important information about the etiology and severity of myocardial injury [54] and plays an important role in the follow-up testing of cardiovascular sequelae in patients discharged from COVID-19. CMR follow-up in 64 patients recovering from the mild disease at home showed that 71% of participants were detected with residual cardiac damage [55]. Clark et al. [56] conducted a case-control study of soldiers recovering from COVID-19, including 50 soldier cases and 50 healthy soldiers, showing that 94% of soldier cases developed cardiovascular symptoms later in recovery, and further long-term CMR follow-up was performed on 4 soldiers diagnosed with myocarditis, of which 1 soldier still showed abnormal CMR with persistent active myocarditis at 7 months of follow-up. Because persistent myocardial involvement, such as myocarditis, can cause adverse consequences such as sudden cardiac death during moderate to high-intensity sports, researchers have followed up competitive athletes with previous COVID-19 and found that 46% of athletes had late gadolinium enhancement (LGE) after CMR testing, and 15% of athletes met the CMR diagnostic criteria for myocarditis [57]. These results have attracted the attention of sports cardiologists and proposed relevant medical evaluation and exercise recovery plans [58].

In a cohort study involving 12095836 patients, 12 months after SARS-CoV-2 infection, it was found that the risk of developing cardiovascular diseases, including arrhythmias, ischemic and non-ischemic heart disease, pericarditis, myocarditis, heart failure, and thromboembolic disease, regardless of age, race, sex, and other cardiovascular risk factors, including obesity, hypertension, diabetes, chronic kidney disease, and hyperlipidemia. These risks are also increased in people who did not have any cardiovascular disease before infection with SARS-CoV-2, indicating that even people without high-risk factors for cardiovascular disease have an increased risk of developing cardiovascular disease after infection with SARS-CoV-2 [59]. In a study focused on myocarditis [60], patients experienced a gradual transition from mild symptoms of fatigue and dyspnea to complete failure. In these patients, elevated troponin and brain natriuretic peptide also reflect myocardial damage and ventricular dilation, but ECG findings are nonspecific and do not show typical diffuse ST-segment elevation [61].

In addition, it has been observed in patients recovering from SARS that long-term lipid metabolism disorders can be caused after treatment with high-dose steroids in the acute phase of the hospital, which is associated with the occurrence of cardiovascular sequelae [62]. In patients with acute COVID-19 infection, glucocorticoid therapy should be given as appropriate to patients with progressive deterioration, and whether it will lead to the occurrence of hormonal therapy-related sequelae needs to be observed over time. Previous studies have confirmed that cases of pneumonia requiring hospitalization for various reasons are risk factors for cardiovascular disease after hospitalization, and the risk of cardiovascular disease can increase by 2~8 times within 1 month after discharge, even if there is still a risk of cardiovascular disease 10 years after discharge [63]. Based on these phenomena, long-term follow-up is critical for patients who have recovered from COVID-19 (Table 2).

**Table 2:** Other damage during follow-up of COVID-19 patient.

Systems	No. of patients	Age and sex	Patient characteristics	Follow-up time	Symptoms	Ref.
Circulatory system	538	52 (41–62) years; female 293 (54.5%)	excluding those with severe and complex underlying diseases or receiving invasive treatment, and who were pregnant or breastfeeding	97 (95–102) days	Cardiovascular-related symptoms (70, 13%), resting heart rate increase 60 (11.2%); discontinuous flushing 26 (4.8%)	[49]
	179	56.5 ± 14.6 years; female 53 (37.1%)	21 (15%) received non-invasive ventilation and 7 (5%) received invasive ventilation	60.3 ± 13.6 days	Chest pain (21.7%)	[2]
	100	49 ± 14 years; male 53 (53%)	67 (67%) recovered at home, 33 (33%) required hospitalization	71 (64–92) days	Abnormal CMR (78), including raised myocardial native T1 (73), raised myocardial native T2 (60), myocardial late gadolinium enhancement (32), or pericardial enhancement (22)	[51]
	1216	62 (52–71) years; male (70%)	60% with a critical care	between 3 and 20 April 2020.	Abnormal echocardiogram 667 (55%), new myocardial infarction in 36 (3%). Without pre-existing cardiac disease (*n* = 901), the abnormal echocardiogram (46%), severe disease (13%)	[52]
	139	52 (41–57) years; female (71.9%)	Healthcare workers	between March 13 and April 25, 2020	Abnormal CMR (60.4%). Either pericarditis and/or myocarditis (30.9%): isolated pericarditis(5.8%), myopericarditis (7.9%), and isolated myocarditis (17.3%)	[53]
	50	Mean 27 years	Soldiers, moderate symptoms (43, 86%), only 10% required hospitalization	71 days	Cardiovascular pathology (12%), myocarditis (8%)	[56]
	26	19.5 ± 1.5 years; male 15 (57.7%)	For Competitive college athletes, no athletes required hospitalization	between June and August 2020	LGE after CMR testing (46%), and myocarditis (15%)	[57]
	1	63 years; male		33 days	Trop I level (up to 11.37 g/L), diffuse myocardial dyskinesia, LVEF decreased	[61]
Nervous system and psychiatric disorders	59	18–79 years; female 29 (49.2%)		between March 3, 2020, and March 29, 2020	Smell and taste loss was reported in 68% (40/59) and 71% (42/59) respectively	[72]
	100	55 (48–65) years; male 57 (57%)	mild (24), moderate (28), and severe (48)	225 (187–262) days	Fatigue (40), “brain fog” (29), and changes in cognition (25)	[77]
	130	53.9 ± 16.4 years; female 66 (50.8%)	Hospitalization, 104 (80.0%)	3-week	headache (97, 74.6%), severe headache (24), anosmia/ageusia (54.6% vs. 18.2% in headache)	[78]
	1	46 years; male	No history of alcohol intake, cigarette smoking, or recreational drug use	53 days	Late onset of Guillain-Barré syndrome	[83]
	402	58 (18–87) years; male 265	Hospitalization (300), managed at home (102)	1-month	28% PTSD, 31% depression, 42% anxiety, 20% OC symptoms, and 40% insomnia	[90].
	1733	57 (47–65) years; male 897 (52%)	68% of participants required oxygen therapy during the hospital, and 7% required HFNC, non-IMV, or IMV; 4% were admitted to ICU; 102 (6%) smokers	6-month	Fatigue or muscle weakness (63%), sleep difficulty (26%), anxiety or depression (23%)	[17]
	62354	49·3 ± 19·7 years; female 34564 (55·4%)		14–90 days	Psychiatric illness (18.1%), psychotic disorder (0.94%), mood disorder (9.9%), anxiety disorder (12.8%)	[91]
	41346	46·6 ± 12·9 years; female 6772 (67·9%)	psychiatric disorders(yes 3110 (31·2%), no 6772 (67·9%))	5.65 ± 4·26 months	Depression (PR 1·18 [95% CI 1.03–1.36]) and poorer sleep quality (1.13 [1.03–1.24])	[92]
Digestive effects	117	60 years or older		90 days	15 (13%) gastrointestinal symptoms on admission, 49 (42%) during hospitalization, and 52 (44%) after discharge	[102]
	1733	57 (47–65) years; male 897 (52%)	68% of participants required oxygen therapy during the hospital, and 7% required HFNC, non-IMV, or IMV; 4% were admitted to ICU; 102 (6%) smokers	6-month	decreased appetite (8%), diarrhea or vomiting (5%)	[17]
Other systems	3993			February 27 to May 30, 2020	1835 (46%) AKI; 347 (19%) AKI patients required dialysis	[110]
	1733	57 (47–65) years; male 897 (52%)	68% of participants required oxygen therapy during the hospital, and 7% required HFNC, non-IMV, or IMV; 4% were admitted to ICU; 102 (6%) smokers	6-month	35% renal insufficiency	[17]
	1250	62 (50–72) years; Male 648 (51.8%)	975 (78.0%) went home, 158 (12.6%) were discharged, 84 patients died	90 days	1.55% risk of VTE, 1.71% risk of arterial thrombosis, and 1.73% major bleeding	[24]
	14	49 (23–64) years; female 12	14 hospitalization, 4 ordinary hospitalization, 2 asymptomatic	5 (1–6) months	Hair loss	[121]

### Effects of PASC on the nervous system and psychiatric disorders

Previous studies have confirmed that ACE2 can be expressed not only in the vascular endothelium, but also in glial cells and neurons [64], so SARS-CoV-2 can cause a series of neurological symptoms through mechanisms such as damage to cerebral circulation, nerve tissue, and its secondary systemic inflammatory response, mainly including taste disorders, smell disorders, headaches, dizziness, etc. [65]. At present, neurological damage is considered to be a more serious hazard after the infection of SARS-CoV-2. One retrospective study found that there were 36.4% of patients have neurological symptoms, which are mainly divided into three categories: one is central nervous system manifestations, such as headache, dizziness, consciousness disorder, acute cerebrovascular disease, epilepsy, poor concentration, memory impairment, and other cognitive dysfunction; the second is peripheral nervous system manifestations, such as loss of taste, loss of smell, loss of appetite, neuralgia, hearing loss, tinnitus, etc.; the third is a skeletal muscle injury, such as muscle pain. Compared with patients with non-severe SARS-CoV-2 infection, patients with severe infection are more likely to have neurological symptoms, especially after acute cerebrovascular disease, impaired consciousness, and skeletal muscle injury. In addition, higher levels of creatine kinase and lactate dehydrogenase have been found in patients with muscle symptoms and severe infection than in patients with non-severe infection [66] (Table 2).

#### Olfactory and taste dysfunction

Olfactory and taste disorders are typical symptoms of COVID-19, and studies have found that more than 50% of patients have smell or taste disorders 4 weeks after the acute phase [67], and some patients (11.7%) can persist up to 1 year after infection [68]. A two-month follow-up found that 11~13.1% of “recovered” patients would lose taste and smell [17]. In addition, studies have found sensory impairments such as hearing loss and tinnitus in cured patients [69]. Psychophysical olfactory testing has found that olfactory dysfunction occurs mainly in patients with severe infections [70]. Olfactory dysfunction can be detected in 21% of patients 40 days after infection [71]. A single-center study from Italy, using 33 standardized psychophysical olfactory recognition tests, showed a 64% incidence of olfactory dysfunction in patients with moderate to severe SARS-CoV-2 infection and 58% in recovered patients with olfactory dysfunction during the acute phase of infection, which is similar to the proportion of patients who developed hypoxemia during hospitalization (64%). The subjective olfactory recovery time of the recovered person varies from a few days to 4 months. Studies [72] have also found that the loss of smell is as high as 68%, suggesting that persistent olfactory loss may be a long-term sequela of SARS-CoV-2 infection.

#### Cognitive changes

Studies have shown that patients who have recovered from COVID-19 have cognitive deficits, mainly manifested as short-term memory impairment, poor concentration, executive function, and visual-spatial processing [73–75]. Severe COVID-19 can cause acute respiratory distress syndrome (ARDS), and studies [76] have found cognitive impairment in patients with ADRS, such as memory impairment (13%), language impairment (16%), and behavioral impairment (49%). This also suggests that PASC patients are likely to have such sequelae. Studies have shown that the levels of biomarkers of central nervous system injury such as plasma neurotrophic factor in the acute phase of infection are abnormally high until they gradually return to normal levels at 6 months of follow-up, but neurological symptoms persist, such as fatigue, brain fog, and cognitive changes [77]. Therefore, there is reason to suspect that neuro-sequelae caused by COVID-19 may not be accompanied by ongoing central nervous system damage, and further comprehensive long-term follow-up of the nervous system is required to clarify the mechanisms underlying the onset of sequelae.

#### Headache

Headache is another persistent symptom of the nervous system after SARS-CoV-2 infection, with up to 91% of recovered patients having intermittent headaches lasting longer than 28 days. A study from Spain showed that 74.6% of patients had headaches during hospitalization, of which 24.7% had severe migraine-like pain attacks, and some patients had a loss of smell and taste. It cannot be ignored that after 6 weeks of follow-up, 37.8% of patients still have a persistent headache, and more than 50% of them have no previous history of headache [78]. Studies have shown a correlation between persistent headache and olfactory or taste dysfunction, which may be related to mechanisms such as SARS-CoV-2-induced hyperinflammation and viral invasion of peripheral nerve endings and damage to the trigeminal vascular endothelium [79].

#### Sensory and motor dysfunction

In addition to other neurological injuries, stroke has been identified as a common disease after severe infection with SARS-CoV-2, which is mainly manifested as sensory dysfunction (such as numbness of the face and limbs, tinnitus, blurred vision, and possibly blindness) and motor dysfunction (such as inflexible limbs, unsteady walking, muscle spasms, etc.), but its pathophysiology is still unclear. Hypercoagulaemia induced by sepsis, as well as endothelial dysfunction secondary to it and the formation of intravascular blood thrombus, may be the cause. At present, in patients infected with SARS-CoV-2, stroke includes venous sinus thrombosis, subarachnoid hemorrhage, cerebral hemorrhage, and ischemic stroke, of which ischemic stroke is the most common [80]. The appearance of stroke symptoms is usually related to large vessel occlusion 1~3 weeks after infection, and the incidence of stroke in men is higher than in women. Compared with stroke patients without SARS-CoV-2 infection, patients with SARS-CoV-2 infection have higher D-dimer, ferritin, lactate dehydrogenase, and troponin, but the incidence of radiographically confirmed ischemic stroke is low, and most strokes are cryptogenic and may be associated with acquired hypercoagulability [81, 82].

The neurotropic nature of SARS-CoV-2 is also manifested in the invasion of the nervous system leading to neuroinflammation and demyelinating changes. Cases have been reported [83] in previously healthy men with leg pain and loss of sensation in their feet 53 days after COVID-19 diagnosis, followed by gradual involvement of extremities, face, and respiratory muscles, and cerebrospinal fluid and nerve conduction studies to support the diagnosis of Guillain-Barré syndrome. A woman with signs of fatigue, intermittent tingling and numbness in the extremities, and blurred vision 3 weeks after infection with COVID-19 showed demyelination on brain MRI and was finally diagnosed with multiple sclerosis caused by COVID-19 after ruling out other causes [84].

#### Hypothalamic-pituitary dysfunction

Previous studies have suggested that SARS-CoV-2 infection can cause the hypothalamic-pituitary axis to be affected, resulting in circadian clock disorders (such as day and night sleep reversal), sexual dysfunction, and diabetes insipidus. In a small number of male convalescent patients, dysfunction of the hypothalamic-pituitary-testicular axis occurs due to immune-mediated injury and sexual dysfunction occurs [85]. Similarly, a 68-year-old man with severe SARS-CoV-2 infection developed pituitary dysfunction, presenting with altered mental status, polyuria, and other features of diabetes insipidus 23 days after infection. Inflammation-mediated reversible hypophysitis and direct immune-mediated injury may be explained by the causes of hypothalamic-pituitary axis involvement following SARS-CoV-2 infection. Since both the hypothalamus and pituitary tissues highly express ACE-2, SARS-CoV-2 could directly target these tissues [86].

#### Abnormal swallowing function

Patients infected with SARS-CoV-2 are at risk of developing dysphagia, which may be associated with peripheral neuropathy of the IX and X cranial nerves following SARS-CoV-2 infection. Ishkanian et al. reported a case of dysphagia related to myositis caused by this virus, while it is known that aspiration, pneumonia, malnutrition, and prolonged hospitalization may contribute to dysphagia [87]. Bell’s palsy, also known as idiopathic facial nerve palsy, is a kind of hypomotor neuron facial palsy of unknown etiology, and a prospective study conducted by HOGG et al. [88] found that the incidence of pediatric Bell’s palsy increased significantly during SARS-CoV-2 epidemic, and studies on adult patients with SARS-CoV-2 infection complicated by facial paralysis have been reported.

#### Psychiatric disorders caused by PASC

Many psychosomatic symptoms have been reported in recovered COVID-19 patients, mainly PTSD, anxiety, depression, and insomnia [20], which are similar to those reported after previous SARS and MERS epidemics [89, 90]. Early follow-up results 1 month after diagnosis of COVID-19 showed a prevalence of psychosomatic symptoms, including anxiety (42%), insomnia (40%), depression (31%), PTSD (28%), and obsessive-compulsive symptoms (20%) [90]. Fatigue or muscle weakness (63%), sleep difficulty (26%), anxiety or depression (23%) were still present when COVID-19 patients were discharged from the hospital at 6 months, with women and severity of disease during the acute infection period being risk factors for persistent psychosomatic symptoms [17]. A large cohort study in the United States showed an increased rate of diagnosis of psychiatric disorders in patients 14–90 days after infection with COVID-19 compared with other disorders (e.g., other respiratory diseases, skin infections, fractures, etc.) [91]. The most recent follow-up reports show that psychiatric symptoms in some patients can persist up to 16 months after discharge [92]. Previous studies on the sequelae of SARS patients have found that mental health disorders and chronic fatigue problems persist for up to 4 years and still affect more than 40% of recovered patients [89]. Therefore, mental health assessment of the recovered COVID-19 population needs to be continued for a long time.

The development of psychiatric disorders associated with COVID-19 involves multiple factors, including biological factors such as immune regulation disorders and cytokine storm caused by SARS-CoV-2 [90], and psychosocial factors such as female, advanced age, economic pressure, social isolation, and fear of disease, which together contribute to the development of psychiatric disorders in rehabilitated patients and seriously affect their quality of life, as well as social development. It has been shown that some recovered patients with COVID-19 are at “moderate risk of suicide“ [93], and 27.60% of patients need psychotropic medication to relieve their psychological disorders within 1 year after SARS-CoV-2 infection [94]. Therefore, it is necessary to provide early primary prevention for people with pre-existing mental illness and identified risk factors, as well as to assess the mental health status of recovered patients as early as possible, so that effective psychological interventions can be actively taken to alleviate the psycho-spiritual stress of the recovered population and promote their full recovery [95].

### Digestive effects of PASC

In a retrospective study by Aiyegbusi et al. [96] of GI symptoms in patients “recovering” from neoconiosis, diarrhea was in the top 10, with a 6% incidence. Other long-term symptoms included nausea, vomiting, abdominal pain, and loss of appetite. Several early studies have confirmed that COVID-19 patients have gastrointestinal symptoms such as loss of appetite, diarrhea, and vomiting during the acute infection period, and that SARS-CoV-2 RNA can be detected in the stool of COVID-19 patients [97–100]. One study reported an overall incidence of gastrointestinal illness of 6% 1 month after diagnosis of COVID-19, including abdominal pain, decreased appetite, diarrhea, and vomiting [101]. At 90 days after infection, up to 44% of patients had gastrointestinal symptoms, mainly loss of appetite (24%), nausea (18%), acid reflux (18%), and diarrhea (15%) [102]. At 6 months of follow-up, gastrointestinal symptoms were still observed, mainly decreased appetite (8%), and diarrhea or vomiting (5%) [17]. The long-term persistence of gastrointestinal symptoms in COVID-19 patients may be related to the prolonged presence of SARS-CoV-2 in the gastrointestinal tract. It has been shown that viral RNA in the stool can be positive even after the respiratory SARS-CoV-2 RNA test is negative, indicating that SARS-CoV-2 can persist in the gastrointestinal tract after respiratory clearance, with a positive duration of 28 days on average and up to 47 days in some patients [98]. In addition, the “enteropulmonary axis”, formed by the two-way interaction of respiratory infection and the intestinal microbial environment, can lead to changes in the intestinal microenvironment in respiratory infections caused by coronaviruses and influenza viruses [103]. As the target organ of SARS-CoV-2, studies have demonstrated that SARS-CoV-2 infection can lead to changes in intestinal flora during acute hospitalization, with an increase in opportunistic pathogenic bacteria and a decrease in beneficial intestinal microorganisms, and intestinal dysbiosis persists even after nasopharyngeal swabs and stool specimens are negative for viral nucleic acid and respiratory symptoms disappear [104]. Therefore, the long-term effects of COVID-19 on the gastrointestinal system need to be studied in depth to better understand the mechanism of the development of persistent gastrointestinal symptoms and to provide more scientific guidance for patients’ recovery.

Abnormal liver function is common in the acute phase of COVID-19 and may result from hepatocellular damage or cholestasis, this is associated with the direct cytotoxicity of viruses, particularly in the biliary tract, as well as systemic inflammation, hypoxia, coagulopathy, and unhealthy drug reactions [105, 106]. However, in patients with COVID-19 with acute liver injury, abnormal liver function may persist for a long time after discharge, and it may take weeks to months before gradual improvement [105] (Table 2).

### Effects of PASC on other systems

#### Effect of PASC on the urinary system

Acute renal injury (AKI) is common in the acute phase of COVID-19, with approximately 5% of hospitalized patients requiring hemodialysis [107]. AKI has many etiologies, including direct viral injury, renal hypoxia, inflammatory cytokine effects, and coagulation abnormalities [108]. AKI is positively associated with in-hospital mortality, and patients discharged from the hospital with PASC may have symptoms of residual renal insufficiency [107]. The main clinical manifestations of acute kidney injury are hematuria, proteinuria, oliguria, and elevated blood creatinine and/or urea nitrogen, 35% of patients with COVID-19 were found to have renal insufficiency at discharge, and up to 30% of patients hospitalized on dialysis for COVID-19 were found to require dialysis support after “recovery” discharge. For AKI patients requiring RRT during hospitalization for acute infection, 41% were discharged with renal improvement and RRT could be discontinued, while 8% required continued RRT [109]. In one study, 36% of patients with concurrent renal disease before discharge were found to be fully recovered, but 14% of these “recovered” patients had a recurrence of renal disease [110]. In another study, it was found that 35% of patients who “recovered” from COVID-19 developed renal insufficiency 6 months after discharge, of which 13% had a previously normal renal function, possibly related to the enrichment of angiotensin-converting enzyme 2 in the proximal tubules of the kidney, which mediates the accumulation of SARS-CoV-2 into epithelial cells, causing cytotoxicity and inflammatory cell infiltration [17]. Therefore, it is necessary to closely monitor the renal function of COVID-19 survivors with renal impairment in the acute stage and new renal insufficiency after discharge, to take appropriate prevention and treatment measures in time to improve their kidney function and reduce the long-term burden of COVID-19.

#### Manifestations of hematologic damage due to PASC

The etiology of this coagulation disorder is multifactorial, including microvascular dysfunction, increased tissue factor expression due to inflammatory cytokines, and the effect of hypoxia on the upregulation of hypoxia-induced transcription factors, especially in critically ill patients [21]. Following SARS-CoV-2 infection, the release of inflammatory factors and the upregulation of tissue factors and thrombin, which cause platelet activation, lead to endothelial cell lesions and a prothrombotic state [82]. Considering the increased risk of thrombosis in patients with this disease, a study comparing the routine use of a medium-dose anticoagulant (enoxaparin 1 mg/kg/day) with a standard prophylactic dose (enoxaparin 40 mg/day) in critically ill patients with COVID-19, the results showed no advantage of medium-dose anticoagulation compared with the standard dose in reducing the incidence of thrombosis, the success rate of extracorporeal membrane pulmonary oxygenation therapy, and the 30-days mortality rate. There was no advantage in reducing the incidence of thrombosis, the success of extracorporeal lung oxygenation, and 30-day mortality compared to standard doses [111]. Hemorrhagic disease also occurs, but given the relatively low risk of major bleeding, inpatient venous thromboembolism (VTE) prophylaxis is beneficial for most patients [112]. In several retrospective studies [112–115], the incidence of VTE in newly discharged patients who had “recovered” from COVID-19 ranged from 0.48% to 1.9%. These VTE rates were similar to those of patients discharged from the hospital after recovery from other diseases [116]. In a large prospective study designed to better assess the probability of various hematologic disorders in patients with PASC, the risk of VTE was found to be 1.55%, the risk of arterial thrombosis was 1.71%, and the risk of major bleeding was 1.73% [24]. Based on these results, the investigators concluded that post-discharge pharmacological thromboprophylaxis could reduce the incidence of death and VTE in patients with PASC [115].

#### Manifestations of skin damage caused by PASC

Hair loss is one of the most common long-term persistent symptoms in patients recovering from COVID-19, with approximately 25% of those followed up having hair loss that lasts longer than 6 months [17, 117]. In addition, skin lesions associated with COVID-19 include measles-like changes as the most common skin manifestation, with an incidence of 36.1%, followed by a maculopapular rash (34.7%) and urticaria (9.7%). Painful erythematous papules on the extremities (15.3%), mainly on the trunk (66.7%), and 19.4% of the skin manifestations on the hands and feet [118]. In a study of “recovered” patients with COVID-19, only 47 of 1655 patients (3%) developed a rash 6 months after infection with the virus [119]. Another study involving 2560 patients [120] found that frostbite-like lesions were the most common cutaneous manifestation (51.5%) and that the incubation period between upper respiratory tract infection and cutaneous manifestation was 1.5 days in children compared to 7.9 days in adults. ROSSI et al. [121] reported resting alopecia in 14 cases of SARS-CoV-2 infection from 1 to 3 months after discharge, earlier than typical resting alopecia, with a median duration of alopecia of 5 months (between 1 and 6 months). However, the trichogram characteristics did not differ from those of typical telogen effluvium, and the possible mechanisms were the release of pro-inflammatory cytokines and direct viral damage to the hair follicles. In a 3-month follow-up of 538 patients with SARS-CoV-2 infection, physical decline or fatigue, post-activity apnea, and hair loss were more common in women than men [122] (Table 2).

## Pathophysiology mechanisms of PASC

The pathophysiological mechanism of PASC is still unclear, but it is generally believed that the persistence of low-load virus, abnormal immune response caused by viral proteins, secondary autoimmune diseases, and multisystem damage caused by microthrombosis and other factors lead to the occurrence and progression of PASC.

### Pathophysiological mechanisms of PASC on the respiratory system

Pulmonary fibrosis following SARS-CoV-2 infection is a major pathological basis for chronic respiratory complications [123, 124]. Studies [125] have discussed the mechanisms that lead to pulmonary fibrosis, which usually occurs after severe respiratory inflammation and injury. In most severe cases, prolonged oxygen may increase oxidative stress in the lungs, thereby maintaining inflammatory status and activating the pulmonary fibrosis pathway. Autopsies of patients who have died of COVID-19 infection have been found to have fibroproliferative lesions, vascular endothelial lesions, and angioregenerative alterations in the lungs [126–128]. Long-term respiratory symptoms can also be due to pulmonary vascular disease. Dhawan et al. [129] suggested that microvascular injury in the lungs may lead to secondary pulmonary hypertension and pulmonary fibrosis. Some studies [130, 131] have pointed out that some patients who do not develop lung lesions after SARS-CoV-2 infection also have dyspnea, and the cause of this is related to the dysfunction of lung ventilation regulation caused by autonomic dysfunction caused by the virus.

Clinically, several studies [132–134] have been devoted to unraveling the underlying mechanisms that lead to long-term lung inflammation. Bai et al. used PET/CT studies [132] to find long-term lung inflammation in patients requiring mechanical ventilation. Chun et al. [133] found that elevated biomarkers of inflammation and fibrosis could be monitored in the blood nine weeks after recovery, regardless of whether patients were hospitalized or not. Sonnweber et al. [134] showed that respiratory disorders are associated with dysregulation of iron metabolism and may be associated with lung damage, particularly in patients with PASC, particularly with pulmonary sequelae.

### Pathophysiological mechanisms of PASC on the cardiovascular system

At present, most researchers believe that SARS CoV-2 mainly causes damage to the heart through the following pathways: (1) SARS CoV-2 directly invades myocardial cells and destroys the renin-angiotensin-aldosterone (RAS) system after binding to ACE2, resulting in myocardial damage [135]; (2) causing endothelial inflammation and microthrombosis in the myocardium, resulting in ischemia and hypoxia of myocardial cells, and secondary damage [136–138]; (3) induce autoantigen-antibody responses and release toxic cytokines to damage cardiomyocytes [139]; and (4) hypoxemia and stress myocardial injury [140].

The invasion of cardiomyocytes through the ACE2 receptor is thought to be a potential mechanism of cardiac dysfunction. In addition, the heart may subsequently undergo structural remodeling due to fibrosis due to long-term inflammatory effects, which may lead to heart failure or arrhythmias [141]. In addition, microvascular endothelial dysfunction in the heart and blood vessels may cause microthrombosis, which can impair the cardiovascular system to varying degrees [141]. Autopsy studies [45, 142] have shown that the virus has invaded endothelial cells and cardiomyocytes, causing signs of vascular inflammation and cardiac dysfunction. Chioh et al. [143] and Sawastano et al. [144] have shown signs of microcirculation disorders in some patients after recovery from COVID-19 infection. Autoimmune disorders are another pathophysiological effect of coronavirus infection. Elevated antiphospholipid antibodies can lead to complications such as vasculitis and blood clots. These antibodies have been found in patients with COVID-19 infection during the acute onset of illness [137, 145]. Sollini et al. [146] used PET/CT to study patients with long-term cardiovascular symptoms after recovering from COVID-19 infection, which conclusively suggested that there is long-lasting inflammation in the large blood vessels. Cardiovascular disease can also be caused by autonomic nervous system disorders. Studies [141] suggest that position orthostatic tachycardia syndrome (POTS) may be caused by viral or autoimmune-mediated receptor dysfunction in the brainstem. Wallukat et al. [139] isolated antibodies to catecholamine receptors that regulate heart rate, as well as antibodies against ACE2 receptors and autoantibodies to endothelin, in the blood of patients with POTS. Silva et al. [147] found that long-term cardiac abnormalities may be secondary to myocardial injury that occurs during COVID-19 infection, or may be due to restricted coronary perfusion or severe hypoxia. However, the mechanism of long-standing cardiac symptoms may also come from virus-mediated microvascular disorders or myocardial inflammation.

### Pathophysiological mechanisms of PASC on the nervous system

The coronavirus spreads to the brain through the nose or bloodstream, which can trigger neuroinflammation. Studies [148] have explored the role of persistent neuritis in symptom flare-up: by activating microglia to elicit an autoimmune response, persistent neuritis may lead to neurocognitive impairment or mental health disorders. In addition, local microthrombosis due to the hypercoagulable state of the blood or mitochondrial failure is also thought to be one of the causes of symptoms. Two studies [149, 150] have reported altered brain structure in some patients with PASC who have recovered from COVID-19 infection based on MRI findings. Among them, Lu et al. [149] highlighted structural changes in several brain regions, including the hippocampus, insular lobes, and olfactory cortex. The volume of gray matter in different brain regions such as the hippocampus and cingulate gyrus is associated with memory or olfactory loss. Qin et al. [150] found a decrease in cerebral cortical thickness in patients with PASC, coupled with alterations in the microstructure of the white matter, as well as a decrease in local cerebral blood flow in the frontal and limbic regions. Sun et al. [151] found that neurological markers in the blood of patients recovering from COVID-19 infection increased compared to healthy people. In addition, the autopsy study by Lee et al. [152] also provides theoretical support for the neuroinflammation hypothesis, in which inflammation of the trigeminal nerve and spinal nerve roots is thought to be associated with headache and physical pain [153], loss of taste and smell is associated with an inflammatory response triggered by viral invasion of neuroepithelial cells in the taste buds and olfactory nerves [154], and autonomic involvement can cause cardiovascular system dysfunction.

Similarly, Versace et al. [155] used electrophysiological techniques to find that GABA can damage cerebral cortical circuits. However, studies that have assessed brain metabolism [148–152, 155–162] have found that patients with a variety of neurological symptoms have low brain metabolism, with symptoms including “brain fog” or olfactory disturbances. Inflammation of the trigeminal nerve caused by PASC, is thought to be associated with headaches and general pain.

Regarding the symptoms of loss of smell and taste in recovered patients, two studies [163, 164] have found that neuroepithelial cells are invaded by the novel coronavirus to produce inflammation, which may be the mechanism of olfactory dysfunction. Histologic examination reveals the presence of coronavirus and inflammation within olfactory neuroepithelial cells [165]. Similarly, biopsy showed that the novel coronavirus persisted in the taste bud cells of the tongue [135].

### Pathophysiological mechanisms of PASC on the immune system

Studies [166] suggest that hyperinflammatory cytokine storm and PASC may originate from mast cell activation syndrome. Studies [18, 167] have also pointed out that long-term immune system activation and the resulting chronic inflammation may lead to autoimmune diseases, such as autoimmune organ dysfunction. Shuwa et al. [168] showed that phenotypic changes in T cells and B cells can affect the long-term immunity of COVID-19 patients, resulting in persistent symptoms, while Perez-Gomez et al. [169] found that dendritic cells (DC) decreased to varying degrees within 7 months after COVID-19 infection, so the severity of residual inflammation after COVID-19 infection can be assessed by identifying the amount of residual DC homing and activation markers involved in the inflammatory response. The presence of novel coronavirus in the gut of asymptomatic infected individuals several months after infection, and the memory B cell response to SARS-CoV-2 evolves in a manner that is consistent with antigen persistence, suggests that residual viral proteins in tissues may underlie sustained immune response activation [170]. Some studies [132, 156] have confirmed that PASC can have persistent immune inflammation, resulting in organ function damage, and researchers have used PET/CT to find signs of hypermetabolism in some patients who have recovered from COVID-19 infection, such as lungs, mediastinal lymph nodes, spleen, and liver. Other studies [75, 171] based on MRI examinations found that some patients who recovered from COVID-19 infection had mild multiorgan impairment (heart, lungs, kidneys, liver, pancreas, and spleen). The relationship between vitamin D and PASC has been studied, but no association has been found between the two [172].

### Pathophysiological mechanisms of PASC on other systems

The gastrointestinal microbiota is essential for establishing immune homeostasis [173]. In patients with COVID-19 who are in remission, gut microbiota dysbiosis can persist for up to one month [174]. Changes in the composition of the gut microbiota in patients with COVID-19 can lead to impaired immune responses related to inflammatory factors and blood markers [175]. In addition, ACE2 has been shown to affect the expression of neutral amino acids in the gut, which in turn regulates the composition of the gut microbiota: reduced proportions of intestinal commensal bacteria with immunomodulatory potential (such as Faecalibacterium prausnitzii, Eubacterium rectale and bifidobacteria) in PASC, thereby regulating local and systemic immune responses [174]. It can be seen that the imbalance of intestinal microbiota may disrupt the body’s immune homeostasis, affect the severity and development of the disease, and promote the occurrence of sequelae of COVID-19.

Glomerular podocytes, proximal tubules, pancreatic β cells, thyroid cells, pituitary gland, and hypothalamus co-express ACE2 receptors and can be directly damaged by SARS-CoV-2 invasion, resulting in renal impairment and endocrine disruption [75, 176]. Microthrombosis secondary to endothelial injury and cytokine storms caused by early inflammatory responses can also lead to indirect damage to these organs [177]. In addition, the rapid spread of SARS-CoV-2, the emergence of a large number of patients with a lack of clinical treatment experience, the uncertainty of efficacy due to lack of experience, and the loneliness and abandonment caused by isolation in the epidemic prevention and control policy will cause mental and psychological problems in some susceptible patients, which in turn will lead to the occurrence of various psychiatric symptoms and functional disorders of physical organs.

## Diagnosis and treatment of PASC

The diagnosis of post-COVID syndrome is based on the history and clinical symptoms and is based on the presence of these symptoms and the exclusion of other diseases. There is no standard treatment for post-COVID syndrome, and immunomodulatory therapy has become a widely used treatment modality in view of the important role played by immune system disorders in the development of the disease. To alleviate organ damage from an overactive immune response, steroid anti-inflammatory drugs are generally preferred in the acute phase, and other types of immunosuppressants may be used in the chronic phase [178, 179]. Plasmapheresis and intravenous immunoglobulin may be considered in some patients with severe symptoms [180]. High viral load in the early stages of infection is one of the important factors leading to PASC, and timely use of antiviral drugs can not only effectively reduce viral load and reduce the risk of severe disease but also help prevent the occurrence of PASC and alleviate the sequelae [181]. When accompanied by respiratory manifestations such as cough and sputum production, anti-inflammatory treatment with budesonide or formoterol can be considered, and symptomatic treatment with compound methoxamine and montelukast can be considered. Histamine antagonists, such as loratadine, can be used to alleviate PASC manifestations such as rhinitis, urticaria, and rashes when abnormal mast cell activation contributes to allergic symptoms [182]. For patients at high risk of post-COVID coagulation disease, anticoagulation may be considered to reduce the risk of thromboembolic events. Coenzyme Q10 has antioxidant and membrane stabilizing effects, and appropriate supplementation of CoQ10 can alleviate oxidative stress and alleviate fatigue and post-exercise discomfort [158]. It has also been reported that food supplements with antioxidant and anti-inflammatory activities, such as vitamin C and hydroxytyrosol, not only help the body defend against viral infections but also improve immunity and prevent long-term fatigue and gastrointestinal problems that occur after COVID-19 infection [183]. Finally, when dealing with mental conditions such as anxiety, depression, and brain fog, it is necessary to actively develop psychological support and cognitive behavioral therapy, and if necessary, drugs such as estazolam or sertraline should be given as appropriate interventions. Exercise therapy, particularly graded aerobic exercise and moderate strength training, has a significant effect on reducing pain, increasing physical strength, improving cardiorespiratory fitness, and improving quality of life [184]. Although the mechanism of exercise therapy for autonomic disorders is not well understood, several studies have demonstrated that appropriate exercise can effectively promote the restoration of normal function of the autonomic nervous system [185]. The timely application of the above treatment measures or adjuvant drugs can help to improve the long-term quality of life of patients to a certain extent, and the specific plan needs to be personalized according to the comprehensive evaluation of the patient’s symptom severity and functional indicators.

## Summary and outlook

This article introduces some of the symptoms that have been observed for a long time in patients with COVID-19, which is temporarily called “long COVID” syndrome, now been changed to “Post-acute sequelae after SARS-CoV-2 infection (PASC)”. Regardless of the severity of the initial disease or the age of the patient, PASC is very likely to affect the quality of life of the patient after “recovery”. This article gives a preliminary description of some of the symptoms of PASC. After SARS-CoV-2 infection, it mainly affects the respiratory, cardiovascular, neurological, psychiatric, urinary, blood, skin, and digestive systems. The most common clinical manifestations of PASC are persistent fatigue, intermittent headache, cough, dyspnea, loss of smell and taste, muscle pain, and other symptoms that may occur memory loss, sleep disorders, chest pain or chest tightness, palpitations, depression and anxiety, nausea, diarrhea, rash, etc. However, the current research on PASC is still immature, and it is still unknown whether the recovered group will have new long-term sequelae in the future. This may be due to its complex and variable symptoms and pathophysiological differences in the disease.

This review summarizes several mechanisms that may be involved in PASC. First, virus-mediated cellular changes associated with neurotropism are thought to be the pathophysiological mechanisms that may help explain PASC. This mechanism can be used to explain loss of smell/taste or other symptoms associated with autonomic nervous system dysfunction. Second, dysregulation of the immune response caused by the persistence of the virus in asymptomatic infections is thought to be a pathophysiological mechanism that may cause symptoms of related diseases, including autoimmune responses, pulmonary fibrosis, or autometabolic disorders. Importantly, some scholars have raised the possibility of organ dysfunction through inflammatory or autoimmune mechanisms. In addition, several studies [135, 165, 170] have hypothesized the existence of persistent and insidious viruses based on the screening and identification of viral strains in several organs following acute coronavirus infection. It can be seen that the pathophysiological mechanism of PASC cannot be singular, it consists of many related system mechanisms intertwined with each other. The findings of Arthur et al. [186] provide more and deeper insights into the pathophysiological interpretation of PASC from the aspects of autoinflammation and immunity. Antibodies against the ACE2 receptor were found in the blood of patients who had recovered from the new coronavirus infection, and at the same time, it was found that the activity of the ACE2 receptor in the patient’s body was greatly reduced. This sequence of responses can lead to an increase in angiotensin II, which induces autoinflammation [186].

On the one hand, future research should be devoted to the study of its pathogenesis, to further improve the health management system of patients with SARS-CoV-2 infection, and to conduct more comprehensive and close follow-up of people recovering from COVID-19 to clarify the potential impact of PASC. On the other hand, it is also necessary to establish a PASC medical assistance plan, improve the mental health service system, and then improve the social support system, and urgently need multidisciplinary intervention to establish a personalized treatment and rehabilitation system for different groups of people such as age, gender, occupation, economic level, and region, to promote the comprehensive recovery of the sequelae groups. Governments and health systems around the world should prepare for the urgent and coordinated long-term global response strategies that are still lacking to address the likely increased burden of disease and long-term health problems caused by the COVID-19 pandemic.
